# Longitudinal Microbiome Investigations Reveal Core and Growth-Associated Bacteria During Early Life Stages of *Scylla paramamosain*

**DOI:** 10.3390/microorganisms12122457

**Published:** 2024-11-29

**Authors:** Yin Fu, Yongxu Cheng, Lingbo Ma, Qicun Zhou

**Affiliations:** 1 College of Fisheries and Life Sciences, Shanghai Ocean University, Shanghai 201306, China; fuyin@ecsf.ac.cn; 2 Key Laboratory of East China Sea and Oceanic Fishery Resources Exploitation, East China Sea Fisheries Research Institute, Chinese Academy of Fishery Sciences, Ministry of Agriculture and Rural Affairs, Shanghai 200090, China; 3 Laboratory of Fish Nutrition, School of Marine Sciences, Ningbo University, Ningbo 315211, China; zhouqicun@nbu.edu.cn

**Keywords:** stage-specific microbiome, machine learning, SourceTracker, growth-promoting probiotics, economically important crab, *Scylla paramamosain*, larviculture

## Abstract

In animals, growth and development are strongly correlated with the gut microbiota. The gut of the economically important marine crab (*Scylla paramamosain*) harbors a diverse microbial community, yet its associations with the surrounding environment, growth performance, and developmental stages remain obscure. In this study, we first characterized stage-specific microbiomes and shifts in the contributions of live feed and water via SourceTracker. We observed decreased microbial diversity and increased priority effects along zoea stages. *Psychobacter* was identified as the core genus, whereas *Lactobacillus* was the hub genus connecting different stages. Second, microbial correlations with various stage-specific growth traits were observed under interventions generating enhanced (probiotic mixture enrichment), normal (control), and reduced (antibiotic treatment) microbiomes. By combining machine learning regression and bioinformatics analysis, we identified four candidate growth performance-associated probiotics belonging to *Rhodobacterales*, *Sulfitobacter*, *Confluentimicrobium*, and *Lactobacillus*, respectively. Our study interpreted the dynamics and origins of the *Scylla paramamosain* zoea microbiome and underscored the importance of optimizing potential probiotics to increase growth performance during early life stages in marine invertebrates for effective larviculture.

## 1. Introduction

The initial colonization of early-life microbiome plays a critical role in larval growth and development, affecting host health at later stages [1]. The advent of next-generation sequencing has dramatically expanded our understanding of the type and number of bacteria in the aquatic microbiome, which has been successfully applied in *Macrobrachium rosenbergii* [2], *Penaeus indicus* [3], *Penaeus monodon* [4], *Sparus aurata* [5], and other aquatic animal larvae.

The composition and function of microorganisms in animals may be influenced by external, internal, or random factors. The external factors that influence microbial composition include nutritional status, environmental conditions, and diet [6], whereas the internal factors include different developmental stages, genotypes, genders, immune statuses, etc. [7,8]. In larviculture, microorganisms living in water, on the surface, or in larvae affect early larval development and provide protection against pathogens. In addition, the microbial composition can be selectively altered by artificial dietary interventions, antibiotic administration, probiotic supplementation, and fecal transplantation [9]. These approaches all have the capacity to highly modulate the composition of the gut microbiota, providing an opportunity to study the effects of different microbiota on host physiology [10]. Since these methods of microbiota regulation do not directly affect host growth and development, the changes in host growth and development that occur because of the abovementioned factors are likely to be directly related to the microbiome.

The association between the host and microbiota profoundly affects tissue growth, development, and maturation, as well as organ differentiation [11]. Microbes help with nutrient digestion and absorption by producing vitamins and enzymes, supplementing vitamins, and inhibiting the growth of harmful bacteria (Wei, Wang et al. 2019 [12]). The relationship between the microbiota and the host is complex and interdependent [13] (Linares, Ross et al. 2016), and changes in the gut microbiota between individuals lead to differences in growth performance [14]. Network studies in mammals have shown that microbial ecosystems and pig growth characteristics are interrelated [15] and that the gut microbiota can regulate feed conversion efficiency in pigs, promoting intestinal metabolism [16]. The growth performance of Chinese turtles is significantly correlated with the ratio of *Bacteroides* to *Firmicutes* [17]. Therefore, the abovementioned correlations provide the possibility of manipulating the microbiome to improve growth and development.

Traditional probiotic screening focuses on cultivable microorganisms that can exhibit antagonistic effects on pathogenic microorganisms in vitro [18] or can improve the aquaculture water environment [19]. Early research on crustacean microbiome function applied simple bioinformatic correlation, statistical models, or just by inference. In crustaceans, crawfish gut microbes were found to be positively correlated with growth [20,21]. The gut microbiota of *Litopenaeus vannamei* has been shown to be correlated with shrimp weight [22]. The gut microbiota of *Penaeus monodon* is closely related to host health [23,24]. Structural equation modeling revealed that changes in the intestinal bacterial community were positively correlated with digestive activity, which affects the growth rate of shrimp, and changes in the microbiota may be responsible for excessive growth or stunting [25]. *Firmicutes* in the guts of Chinese mitten crabs are negatively correlated with their length, width, and weight [26]. In mangrove aquaculture, the gut microbiota of the mud crab are constrained via water quality variables, which in turn affect the weight of the crabs [27]. However, owing to the broad functionality of probiotics, the latest research on probiotics has utilized multi-omics data analysis and computer prediction of microbial characteristics to screen for probiotics with specific functions to accurately identify the different probiotic potentials of bacterial strains [28]. Machine learning utilizes large datasets to identify, classify, and predict patterns. In microbiome research, machine learning has been applied to solve tasks such as phenotype analysis (i.e., predicting environmental or host phenotypes), microbial feature classification (i.e., determining the abundance, diversity, or distribution of microbial communities), studying complex physical and chemical interactions between microbial community components, and monitoring changes in microbial community composition.

The mud crab (*Scylla paramamosain*) was originally distributed in the tropical Indo-Western Pacific region and is considered one of the most important economic crabs in Southeast Asia [29], with strong adaptability, fast growth rate, large size, delicious meat, abundant nutrition, and thus high economic value. According to the 2024 China Fisheries Statistical Yearbook, the area of the mud crab aquaculture in China was 24,809 hectares in 2023, with an increase of 2.64% compared to 2022; the total aquaculture output reached 157,012 tons, with an increase of 1.52% from 2022, making it the highest aquaculture product of the marine crabs in China. Mud crab larvae have frequently been in short supply in the industry. Fortunately, the use of probiotics as alternatives to antibiotics has been shown to be promising in aquaculture, particularly in crustacean larviculture [30]. When there are more beneficial rather than pathogenic species, the health and growth of fish or crustaceans can be significantly improved [31]. At present, an understanding of the establishment and maturation of the microbial community in *Scylla paramamosain* larvae is lacking. According to the life cycle, *Scylla paramamosain* first undergoes five zoea stages (Z1–Z5) before the megalopa and the crablet stage, during which the important physiological/behavioral changes such as mouth opening, change in food preference, predation behavior, and immune development greatly influence the establishment of microbiome. Only the proportions of the microbial composition in the breeding water and feed of zoea stage I were revealed by Li et al. [32]; the dynamics of microbial community establishment from other developmental stages were not analyzed, and the effects of different types of live feeds on the microorganisms in larvae were not compared. There is no research yet on the relationship between larval growth performance and the gut microbiota across developmental stages. Moreover, potential probiotics to improve the efficiency of mud crab larviculture warrant further exploration.

Therefore, our study aimed to analyze the characteristics of the microbial communities during the development process of *Scylla paramamosain* zoea, and to establish the relationship between the bacterial community and larval growth performance. We first tracked the dynamics of the microbial community structure of the mud crab zoea at all stages. Second, we analyzed the composition and diversity of the zoea, water, and feed microbiotas throughout the developmental stages to evaluate their contributions via SourceTracker. Third, we applied two microbial intervention models: a commercial probiotic mixture (PM) treatment group containing more than 80 live bacterial species and an antibiotic (Abx) treatment group. On the basis of the correlation between bacterial abundance and growth performance indicators, machine learning combined with traditional bioinformatic analysis was used to screen growth performance-related candidate probiotics in two key developmental stages, zoea III and zoea V, of mud crab larvae.

## 2. Materials and Methods

### 2.1. Ethics Statement

All animal experiments in this study were conducted in accordance with the relevant national and international guidelines. Our project was approved by the East China Sea Fisheries Research Institute. In China, catching wild mud crabs in seawater does not require specific permits. Our study did not involve endangered or protected species. This study used a minimum number of crab larvae without affecting the breeding of the next generation, and minimized the harm to larvae by immediate killing before grinding.

### 2.2. Study Animals

This experiment was conducted in April 2023 at the Zhejiang Ninghai Experimental Base of the East China Sea Fisheries Research Institute in Ninghai Town, Zhejiang Province, China (29.2° N, 121.5° E). A reverse osmosis device was applied for water treatment by removing environmental contaminants including both organic (e.g., pesticides and microplastics) and inorganic contaminants (e.g., potentially toxic elements such as lead, cadmium, arsenic, and selenium), and therefore, the sea water used was stable and safe for crab breeding. Only female, uninjured egg-bearing mud crabs (*Scylla paramamosain*) with the same hatching date were selected, held in dark aquariums with disinfected seawater without the addition of antibiotics/probiotics, and fed ad libitum on razor clams until hatching. The newly hatched larvae were immediately transferred to an 80-cubic-meter seedling pond, and zoea I larvae produced by different mother crabs (1,000,000–2,000,000 zoea I larvae per mother crab) were cultured in separate ponds. The nursery pool used disinfected natural seawater, continuous oxygenation, sufficient sunlight at a salinity of 28–30‰, and a water temperature of 25–27 °C. The rotifers and Artemia were harvested fresh and fed daily. In this study, zoeae were selected as the experimental animals. The zoea of the mud crab can be divided into five developmental stages: zoea (Z) in stages I (Z1), II (Z2), III (Z3), IV (Z4), and V (Z5).

### 2.3. Animal Trial 1 (Establishment of the Zoea Microbiota)

Zoea larvae produced by female crabs on the same day were included in this experiment. The Z1–Z5 produced by each female crab were sampled separately; therefore, we collected samples of different genotypes of larvae at each stage. By observing the morphology of the larvae under a microscope, the developmental stage of the larvae was determined according to Zeng et al. [33]. On the second day after 100% metamorphosis of Z1–Z5 in the nursery pond, 200 larvae from three different female crabs at each stage were randomly scooped from each pond (*n* = 600). The sample size was chosen according to other crustacean larvae experiments [2,3,34]; pooled larval samples of 30 to 200 crustacean zoea were sufficient for DNA extraction, and this study chose the largest sample size of 200 to ensure successful DNA extraction for further analysis. Owing to the small size of the larvae, it was difficult to pick their intestines; thus, according to Niu et al. [35], this study used sterilized tweezers to pick them up and rinse them in sterile distilled water. After being rinsed with 50 mL of 1% benzalkonium chloride solution for 30 s, the larvae were rinsed three times with sterile distilled water. After the bacteria on the larval surface were removed, the larvae were transferred to sterile 1.5 mL centrifuge tubes, each tube was filled with 100 μL of Tris EDTA buffer, and the homogenate was ground with an electric grinder. Moreover, a total of 15 water samples were collected for each stage, Z1–Z5, on the second day after 100% metamorphosis. Five hundred milliliters of nursery water from each pond was sampled after filtering out the zoea and live feed, followed by vacuum filtration and collection via a 0.22 μm polyphenylene ether sulfone membrane. The membrane was folded and stored in a 5 mL sterile Eppendorf tube. The rotifers and Artemia were also rinsed and collected. Three replicates of 10 mL each of rotifers and Artemia were filtered from the rotifer pool and Artemia pool, respectively, and only live feeds were transferred to sterilized centrifuge tubes.

### 2.4. Animal Trial 2 (Microbial Intervention)

Larvae hatched by the same mother crab were selected and divided into nine 5-cubic-meter pools, which were randomly assigned to three PM treatment groups, three Abx treatment groups, and three control groups. Previous studies on *Scylla serrata* larvae proposed that a stocking density of 30 individuals per liter during the zoeal stage was suitable for scientific observations [36,37], and thus, 150,000 zoea I was initially placed for each treatment group to ensure a sufficient sample size for further analysis. In this study, antibiotic and probiotic mixtures were used to induce changes in the microbiome of the zoea while sufficient feeding was maintained. The specific operation involved filtering out the rotifers and Artemia and then soaking 1g in 10mL the original concentration of commercial probiotic mixture (PM) or antibiotics (Abx) for 2 h every other day before feeding. The control group was fed normally cultivated rotifers and Artemia. The commercial probiotic mixture used was purchased from Jiangsu Hengtai Environmental Protection Technology Development Co., Ltd. (Wuxi, China), at pH 3.0–4.0, with a lactic acid bacteria count of 1.0 × 10^7^–1.0 × 10^8^ CFU/mL, a yeast count of 1.0 × 10^4^–1.0 × 10^5^ CFU/mL, a photosynthetic bacteria count of 1.0 × 10^3^–2.0 × 10^3^ CFU/mL, and an actinomycete count of 1.0 × 10^3^–3.0 × 10^3^ CFU/mL, with over 80 viable bacterial species and over 10% metabolites. The antibiotic used was florfenicol, with a concentration of 10 ppm in the medicinal bath. As the most commonly used antibacterial drug in aquaculture worldwide, florfenicol irreversibly binds to the receptor site of the 50S subunit of bacterial ribosomes, blocking peptidyl transfer, inhibiting peptide chain elongation, interfering with protein synthesis, and producing antibacterial effects, which greatly reduces the microbial community in larvae. We used florfenicol only for scientific research experiments, which was strictly prohibited from being used for clinical, medical, or other purposes.

Z3 and Z5 are two key developmental stages of *Scylla paramamosain* zoea. On the second day after 100% metamorphosis of the zoea into Z3 and Z5, ten Z3/Z5 larvae were randomly scooped from each pond, with 3 replicates in each treatment group (*n* = 600), picked with sterilized forceps, washed in sterile distilled water, and placed in a sterilized 1.5 mL centrifuge tube for freezing at −80 °C.

### 2.5. DNA Extraction and Sequencing

A bacterial DNA extraction kit (OMEGA, Norcross, GA, USA, D3350) was used to extract DNA from the grinding solution according to the operating instructions to obtain microbiota DNA. The concentration and purity of the extracted DNA were determined via a NanoDrop 2000 ultramicro spectrophotometer (Thermo Fisher Scientific, Wilmington, NC, USA), and the sample DNA was stored at −35 °C for future use.

The PCR reagents used for PCR amplification included predenaturation at 98 °C for 30 s, denaturation at 98 °C for 10 s, annealing at 54 °C for 30 s, and extension at 72 °C for 45 s, for a total of 32 cycles, and a final extension at 72 °C for 10 min. The following universal primers were used for the bacterial V3-V4 regions: 341F (5′-CCTACGGGNGGCWGCAG-3′) and 805R (5′-GATACHVGGGTATCTAATCC-3′) [38]. The PCR product was purified with AMPure XT beads (Beckman Coulter Genomics, Danvers, MA, USA) and quantified with a Qubit instrument (Invitrogen, Waltham, MA, USA).

The purified PCR products were evaluated via an Agilent 2100 Bioanalyzer (Agilent, Santa Clara, CA, USA) and an Illumina Library Quantification Kit (Kapa Biosciences, Woburn, MA, USA). The qualified library concentration should be above 2 nM. The qualified sequencing libraries (index sequences cannot be duplicated) were diluted in a gradient, mixed according to the required sequencing amount in the corresponding proportion, and denatured into single chains with NaOH for sequencing. Two × 250 bp double-ended sequencing was performed via a NovaSeq 6000 sequencer with a NovaSeq 6000 SP Reagent Kit (Illumina, San Diego, CA, USA) (500 cycles).

### 2.6. Microbiome Data Analysis

The double-ended data are concatenated and filtered to obtain high-quality CleanData. DADA2 [39] (divisive amplitude denoising algorithm) no longer clusters on the basis of sequence similarity but instead uses steps such as clustering on the basis of 100% similarity to obtain representative sequences with single-base precision, improving data accuracy and species resolution. The core of DADA2 is denoising; then, the concept of amplitude sequence variants (ASVs) is used to construct an operational taxonomic unit (OTU) table, and the final ASV feature table and feature sequence were obtained. SILVA (Release 138, https://www.arb-silva.de/documentation/release-138/, accessed on 1 September 2023) and the NT-16S database were used to ensure complete and accurate annotation. The annotation threshold was a confidence level greater than 0.7. Based on the ASV annotation results and the ASV abundance tables, species abundance tables at the kingdom, phylum, class, order, family, genus, and species levels were obtained. Normalization was used to homogenize data distribution prior to statistical analysis and machine learning analysis. Min-Max Normalization was used, which can scale data to a specified range of [Min, Max]. The calculation is Normalized Value = (Value − Minimum Value)/(Maximum Value − Minimum Value), which effectively simplified, redistributed, and standardized the raw data while maintaining data accuracy and reliability. QIIME was used to calculate alpha and beta diversities. PCoA based on the Bray–Curtis distance matrix was performed via R. The R language was also used to perform SourceTracker to analyze the possible sources of microbial communities as well as the proportions of these microbial sources. Microbial SourceTracker analysis [40] is a Bayesian method created by Knights in 2011 that estimates the proportion of pollution in the target environment from the source environment more accurately than other methods do. The Spearman correlation coefficient, which uses a monotonic equation to evaluate the correlation between two statistical variables, performs a rank transformation on variables X and Y, and the correlation between RX and RY was calculated using Pearson correlation analysis. If there are no duplicate values in the data and when two variables are completely monotonically correlated, the Spearman correlation coefficient is +1 or −1.

Machine learning is the process of allowing computers to observe large amounts of data and training them to acquire the ability to analyze and solve problems. This study developed a regression-based random forest model to analyze data from all treatment groups to identify characteristic microorganisms associated with survival rates. Using the randomForest package (Version 4.6–12) in R (4.1.2), the model parameters were set as follows: data preprocessing as the norm, the training set ratio at 0.8, the number of decision trees at 100, the node splitting standard as squared_error, the minimum number of samples for node splitting at 2, the minimum number of samples for leaf nodes at 1, the maximum depth of the tree as not limited, the maximum number of features limited to auto, whether to put back the sampling as Yes, and whether to perform out-of-bag data testing as Yes.

LEfSe (LDA effect size) was used to identify biomarkers with significant differences in abundance between different treatment groups. The Kruskal–Wallis rank sum test was used to identify significantly different species. The Wilcoxon rank sum test was then used to detect whether all subspecies of the significantly different species converged to the same classification level. Finally, linear discriminant analysis (LDA) was used to obtain the final significantly different species.

Using IBM SPSS Statistics V26.0, nonparametric Kruskal–Wallis rank sum test analysis was performed on alpha diversity and differential bacterial genera, and the least significant difference (LSD) test was used for multiple comparisons. Spearman correlation coefficient analysis was performed on the correlations between microbial communities. The significance level was set at *p* < 0.05.

### 2.7. Growth Performance Data Analysis

The survival rate, developmental speed, developmental synchrony, weight, and tolerance of larvae are good indicators of their growth and development performance. In each treatment group, four 5 L beakers were taken daily from each of the three breeding pools after dusk, so 12 replicates were used for counting the number of larvae in each treatment group. The survival rate, larval stage index (LSI), and molting synchronicity index (MSI) were calculated. At the end of the experiment, when all treatment groups were transformed to Z5, four 250 mL paper cups were taken from each pool, which contained 200 mL of water and five Z5, so each treatment group had 12 replicates for evaluating tolerance. The reduction in the number of larvae in the next stage caused by continuous sampling had a negligible effect on the survival rate of each pond.

1. Survival rate

The survival rate of each stage was the percentage of fully metamorphosed zoea that survived after dusk on the first day of each developmental stage compared with the initial number of Z1 larvae as estimated from the beaker.

2. Larval stage index (LSI)

A microscope was used to identify the developmental stage of each juvenile in the beaker, which was assigned values of Z1 = 1, Z2 = 2, etc. [41]. The following formula was used to calculate the daily LSI: LSI = ((A1 × A2) + (B1 × B2))/C, where A1 is the number of larvae remaining in the previous stage, A2 is the value assigned to the previous developmental stage, B1 is the number of larvae in the latest developmental stage, B2 is the value assigned to the latest developmental stage, and C is the total number of larvae.

3. Molting synchronicity index (MSI)

The MSI was calculated as the average of the number of larvae at the previous stage and the number of larvae successfully metamorphosed [36].

4. Final weight

At the end of the experiment, when all the samples were transformed into Z5 (day 17), samples were taken 4 times from each pool, with fifty Z5 samples taken each time. The larvae were rinsed with distilled water, dried in a 40 °C oven for 24 h, and weighed to the nearest 0.1 mg on a microbalance to calculate the individual dry weight (mg/ind).

5. Tolerance
  - Starvation test: On the 17th day after the experiment, twenty Z5 larvae were taken from each pool and placed in four 250 mL paper cups without food. The degree of death was checked every 6 h, and dead individuals were removed until all the larvae died. The standard of death is that there is no response when a soft needle is used to move the appendages. At the end of the experiment, 50% of the time to death was calculated as PNR50 (point of no return when 50% mortality occurred).
  - Osmotic stress test: On the 17th day after the experiment, twenty Z5 larvae were taken from each pool in 28–30‰ seawater and placed in four 250 mL paper cups. The paper cups contained 200 mL of fresh water with a salinity of 0‰. The number of dead individuals was recorded every 3 min until all the larvae died, and the average cumulative number of deaths was calculated within each time interval, known as the cumulative stress index (CSI) [42].

Using IBM SPSS Statistics V26.0, Levene’s test was conducted on the relevant growth indicators of each treatment group, and it was found that the variance was uniform. *t*-tests and one-way ANOVA were used to analyze growth performance indicators, with the least significant difference (LSD) test as the multiple comparison method. The Pearson correlation coefficients between candidate microbial communities selected by random forests and growth performance indicators (survival rate, LSI, MSI, final body weight, PNR50, and CSI) were calculated and plotted via R. The statistical significance level was set at *p* < 0.05.

## 3. Results

Overall, the microbiome of *Scylla paramamosain* zoea consisted of 25 phyla from 777 genera. After quality control, a total of 3,661,044 valid sequences were obtained from the raw sequencing data, with an average of 67,818 ± 5550 valid sequences per sample and ≥Q20 greater than 96.1%, indicating high reliability of base recognition. The rarefaction curves in Figure S1 showed that when the number of sequences reached 12,000, all the dilution curves flattened, indicating that the sequencing depth was able to comprehensively reflect the species composition of the microbiota.

### 3.1. Microbiome Diversity of Zoea, Water, and Live Feed in Scylla paramamosain Larviculture

**Figure 1:**
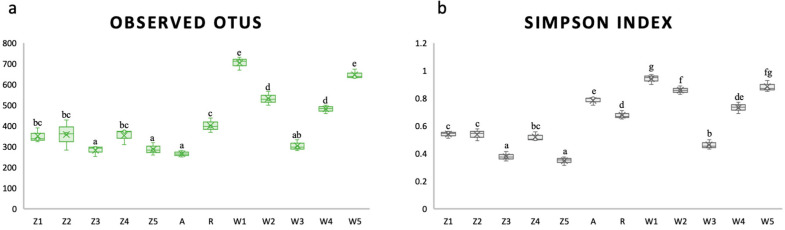
Alpha diversity represented by (**a**) observed OTUs and (**b**) Simpson indices in *Scylla paramamosain* larviculture. Abbreviations are zoea stages I (Z1), II (Z2), III (Z3), IV (Z4), and V (Z5), rotifers (R), artaemia (A), and water (W1–W5) in five different stages of the mud crab zoea larvae. The number of OTUs determines species richness, whereas the Simpson index determines species diversity. Different letters above the box represent *p* < 0.05, denoting a statistically significant difference. *n* = 600.

The dynamics of the microbiomes of *Scylla paramamosain* zoea (Z1–Z5), water (W1–W5) in five stages, and live feed rotifers (R) and Artemia (A) were analyzed via alpha diversity (Figure 1). In terms of live feed, the number of OTUs in rotifers was significantly greater than that in Artemia, but the Simpson index of Artemia was significantly greater than that of rotifers (*p* < 0.05). In terms of water, the number of OTUs and Simpson index from high to low were W1 > W5 > W2 > W4 > W3 (*p* < 0.05). From Z2 to Z4, there was a similar pattern of variation in the alpha diversity of zoea and water. Overall, the number of OTUs in water was greater than that in zoea and live feed, and the Simpson index of water and live feed was greater than that of zoea.

### 3.2. Contributions of Water, Live Feed, and Priority Effect to the Host Microbiome

**Figure 2:**
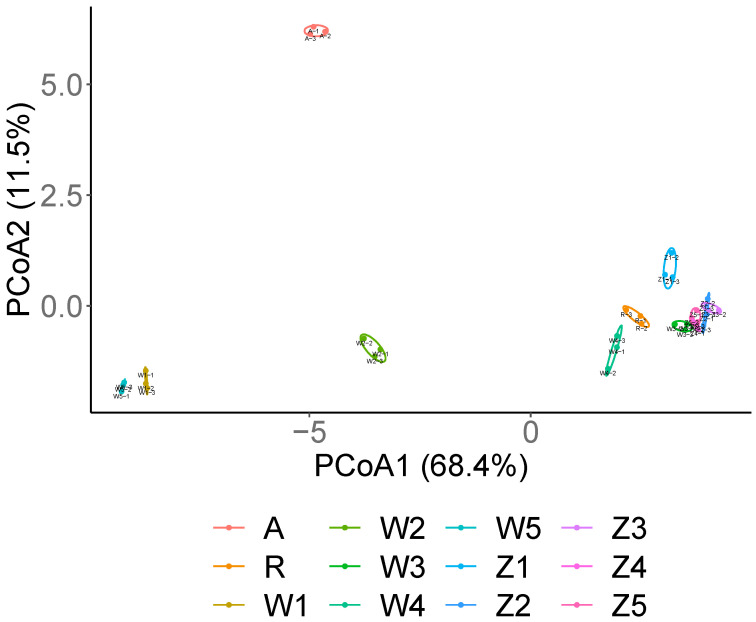
Principal coordinate analysis based on the Bray–Curtis distance in *Scylla paramamosain* larviculture. Abbreviations are zoea stages I (Z1), II (Z2), III (Z3), IV (Z4), V (Z5), rotifers (R), artaemia (A), and water (W1–W5) in five different stages of the mud crab zoea larvae.

Shifts in community membership reflected by beta diversity (Figure 2) indicated that the microbial structure of rotifers, as opposed to that of Artemia, was more similar to that of zoea. Water samples W3 and W4 were similar, W1 and W5 were similar, and W2 diverged from the other four periods. Water W3 was most similar to Z3 larvae, whereas W1, W2, and W5 were distinct from the larvae. Z2–Z5 were relatively close, except for Z1, which was far from the other four stages.

**Figure 3:**
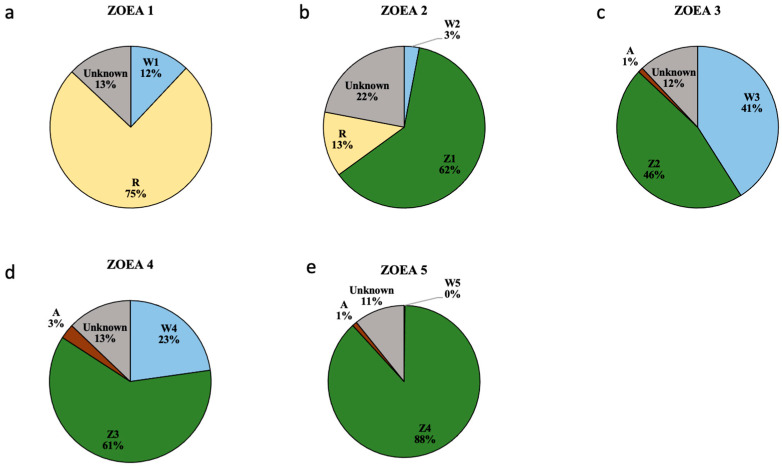
SourceTracker analysis of *Scylla paramamosain* zoea microbiome. Zoea stages (**a**) I (Z1), (**b**) II (Z2), (**c**) III (Z3), (**d**) IV (Z4), and (**e**) V (Z5) are sourced from hosts at the previous stage, rotifers (R), artaemia (A), and water (W1–W5) at five different stages of the mud crab zoea larvae. The pie chart displays the average proportions of 1000 calculations.

The factors shaping the stage-associated zoea microbiome were evaluated via SourceTracker analysis (Figure 3). The microorganisms in the newly hatched Z1 population were mainly rotifers (75%), with rather small contributions from water (12%) and 13% from unknown sources. The priority effect played a role in Z2–Z5, whose microbiome largely originated from the previous stage, accounting for 62%, 46%, 61%, and 88%, respectively. As the zoea developed, an increasing proportion derived from earlier stages was indicative of the stabilization of the internal microbiota. In terms of live feed, the impact of rotifers on the Z1–Z2 microbiota was significantly greater than that of artemia on Z3–Z5, with 75% of the microbiota in Z1 and 13% in Z2 sourced from rotifers and only 1–3% in Z3–Z5 from Artemia. W3 had the greatest impact (41%) on the microbiome in Z3, followed by W4 (23%) in Z4; W2 (3%) in Z2 and W5 accounted for almost 0%, suggesting that the influence of water on the larval microbiome gradually weakened. In addition, unknown sources of microorganisms were present in each stage from 11 to 22%.

### 3.3. Core and Stage-Associated Taxa of Zoea Microbiome

**Figure 4:**
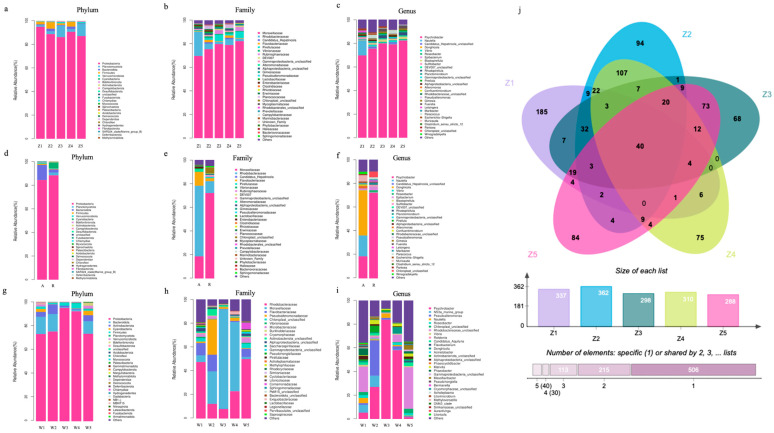
Microbiome compositions of *Scylla paramamosain* zoea, live feed, and water. Stacked bar charts showed top 30 of zoea stages I (Z1), II (Z2), III (Z3), IV (Z4), and V (Z5) at the (**a**) phylum, (**b**) family, and (**c**) genus levels, of rotifers (R) and artaemia (A) at the (**d**) phylum, (**e**) family, and (**f**) genus levels, and of water (W1–W5) at five different stages zoea at the (**g**) phylum, (**h**) family, and (**i**) genus levels. Venn diagram (**j**) of amplicon sequence variants (ASVs) showed that 40 species were in common throughout Z1–Z5.

Figure 4 presents the microbial abundance of the genus at the phylum, family, and genus levels. *Proteobacteria* was the most important phylum, with an abundance ranging from 86.29% to 95.07%, followed by *Planctomycota*, which ranged from 1.44% to 10.20%; *Bacteroides*, which ranged from 0.48% to 2.11%; and *Firmicutes*, which ranged from 0.12% to 5.57%. There were two dominant families, with *Moraxellaceae* accounting for 69.67% to 79.77% and *Rhodobacteraceae* accounting for 2.11% to 20.59%. *Psychobacter* was the predominant genus, with a relative abundance ranging from 69.66% to 82.25%. The core microbiota of the zoea consisted of species shared among multiple stages (Figure S2). The core species with high abundance (Z value > 0) present in all stages included three strains of *Psychobacter* spp., *Fuerstia* unclassified, *Pirellula* unclassified, *Vibrio harveyi*, uncultured *Roseobacter* sp., *Gammaproteobacteria* unclassified, *Blastopirellula* unclassified, *Rhodopirellula* unclassified, DEV007 unclassified, *Gimesia* unclassified, *Muricauda* unclassified, and *Confluentimicrobium* unclassified. Among these genera, the species belonging to *Psychobacter* presented the most consistent abundance at all stages, with high Z values and high intergroup similarity. Therefore, the *Psychobacter* genus was both the dominant and the core genus of *Scylla paramamosain* zoea.

**Figure 5:**
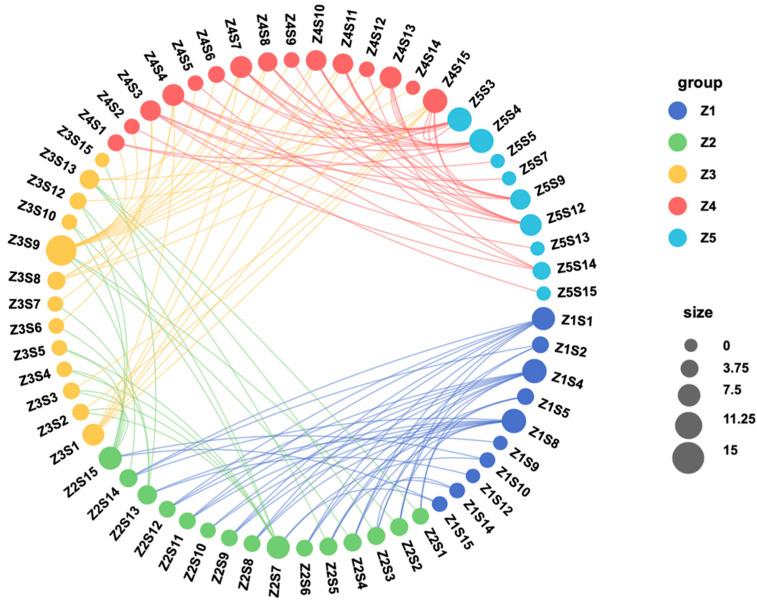
Network topology of Spearman correlation analysis. Different strains of S1–15 in *Scylla paramamosain* zoea stages I (Z1), II (Z2), III (Z3), IV (Z4), and V (Z5) at the species level. The Spearman correlation analysis thresholds were absolute values greater than 0.8 and *p* values less than 0.05. Nodes represent different bacterial species, edges represent significant correlations between nodes, connectivity is represented by node size, and node color represents the classification of different stages of Z1–Z5.

Figure 5 showed the network topology of bacterial species between stages. From Z1 to Z2, Z1S4 (*Leisingera daeponensis*) and Z1S8 (*Epibacterium* unclassified) were significantly correlated with nine bacterial species in Z2. From Z2 to Z3, Z2S7 (*Escherichia–Shigella* unclassified) and Z2S15 (*Lactobacillus* sp.) were significantly correlated with the bacterial species in Z1 and Z3, respectively. From Z3 to Z4, Z3S9 (*Ruegeria atlantica*) was significantly correlated with the bacterial strains in Z2 and Z4. From Z4 to Z5, Z4S15 (*Lactobacillus* sp.) was significantly correlated with the bacterial species in Z3 and Z5, whereas Z5S3 (uncultured *Psychrobacter* sp.) and Z5S4 (*Leisingera daeponensis*) were significantly correlated with nine bacterial species in Z4. Therefore, the strains of the genera *Escherichia-Shigella* and *Lactobacillus*, as well as *Ruegeria atlantica*, might be the hub nodes connecting the different developmental stages of *Scylla paramamosain* zoea.

### 3.4. Effects of Microbial Intervention on Larval Growth Performance

Next, we performed microbial intervention in a second trial through live feed bio-encapsulation. The encapsulated PM in both live feeds were reflected by taxonomy changes shown in Figure S3. The alpha diversity data in Figure S4 indicated that the richness and diversity of the Abx group in both Z3 and Z5 were significantly lower (*p* < 0.05), whereas the richness and diversity of the PM group were lower than those of the control at Z3 and higher at Z5 as the zoea developed. The richness and diversity of the PM group were significantly greater at Z5 than at Z3 (*p* < 0.05). The PCoA results shown in Figure S5 indicated that Z3 in the PM group was similar to that in the control group, whereas Z5 in the PM group was distinct from that in the control group. Z3 and Z5 in the Abx treatment group, however, were far from those in the other treatments.

**Figure 6:**
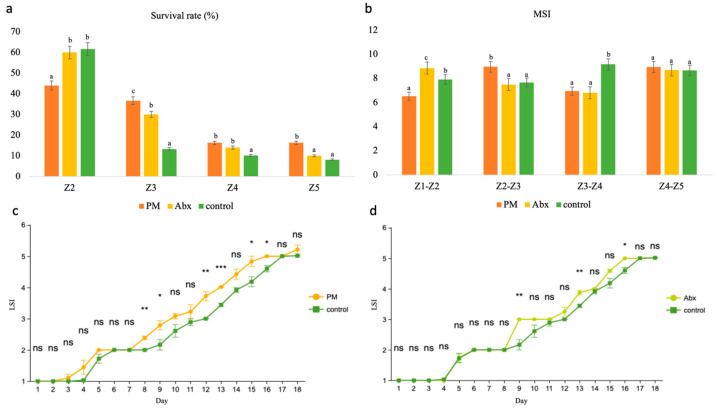
Growth and development of *Scylla paramamosain* zoea affected by microbial intervention. Antibiotics (Abx) and a probiotic mixture (PM) are applied. Growth performance is indicated by (**a**) survival rates and (**b**) molting synchronicity indices (MSIs) of zoea stages I (Z1), II (Z2), III (Z3), IV (Z4), and V (Z5) and daily changes in larval stage indices (LSIs) of zoea affected by (**c**) PM and (**d**) Abx. For (**a**,**b**), different letters above the bars represent *p* < 0.05, denoting statistically significant differences. For (**c**,**d**), * *p* < 0.05; ** *p* < 0.01; *** *p* < 0.001; ns meaning not significant. *n* = 12.

Figure 6 presented altered larval growth performance owing to altered microbiomes. Although the survival rate of Z2 in the PM group was lower than that in the Abx and control groups, the survival rates of Z3, Z4, and Z5 in the PM group (36.67%, 16.32%, and 16.32%, respectively) were significantly greater than those in the Abx and control groups. Compared with the control treatment, the PM treatment also resulted in a greater LSI, and the developmental process was significantly faster than that of the control on days 8, 9, 12, 13, 15, and 16. Moreover, Abx-induced larval development was significantly faster than that in the control on days 9, 13, and 16. In terms of molting synchronicity, each stage showed different patterns, and no significant difference was found from Z4 to Z5.

**Figure 7:**
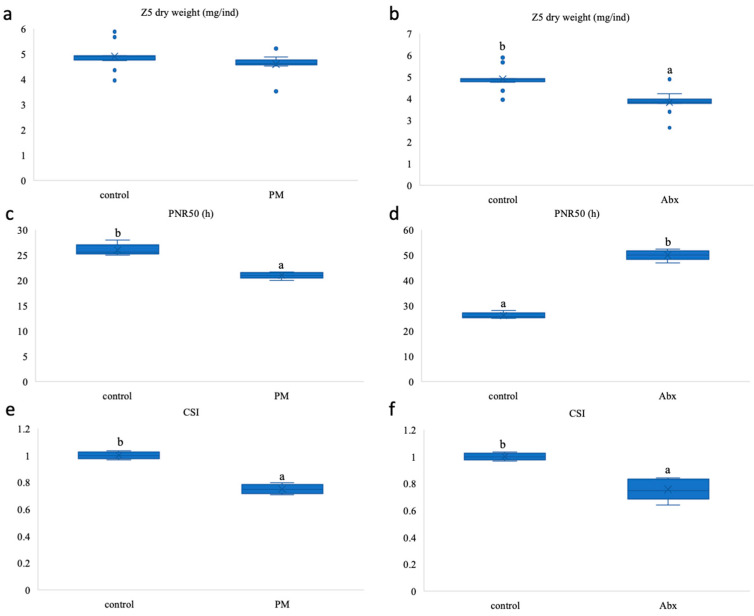
Larval quality of *Scylla paramamosain* zoea stage V on day 17 after hatching. Larval quality is indicated by (**a**,**b**) individual dry body weights; (**c**,**d**) points of no return when 50% mortality (PNR50) occurs in the face of starvation; and (**e**,**f**) cumulative stress indices (CSIs) when saltwater with salinities of 28–30‰ is suddenly transferred to freshwater with a salinity of 0‰, as affected by a prebiotic mixture (PM) and antibiotics (Abx), respectively. Different letters above the box represent *p* < 0.05, denoting a statistically significant difference. *n* = 12.

Figure 7 compared the larval quality between treatments. The individual dry weight of Z5 did not significantly differ between the PM treatment and the control, yet that of Abx was significantly lower than that of the control (*p* < 0.05). With respect to stress tolerance, the hunger tolerance of the Abx group was significantly greater than that of the control group, whereas the hunger tolerance of the PM group was significantly lower than that of the control group (*p* < 0.05). When the zoea were suddenly transferred from seawater to freshwater, the CSI (indicative of sensitivity to freshwater) of the PM and Abx groups was significantly lower than that of the control, which suggested that the two treatments resulted in significantly greater tolerance to sudden salinity drops (*p* < 0.05).

Therefore, the PM treatment group presented the best survival rate, developmental speed, and tolerance to osmotic stress, whereas the Abx group presented the best tolerance to hunger.

### 3.5. Growth Performance-Associated Bacteria

**Figure 8:**
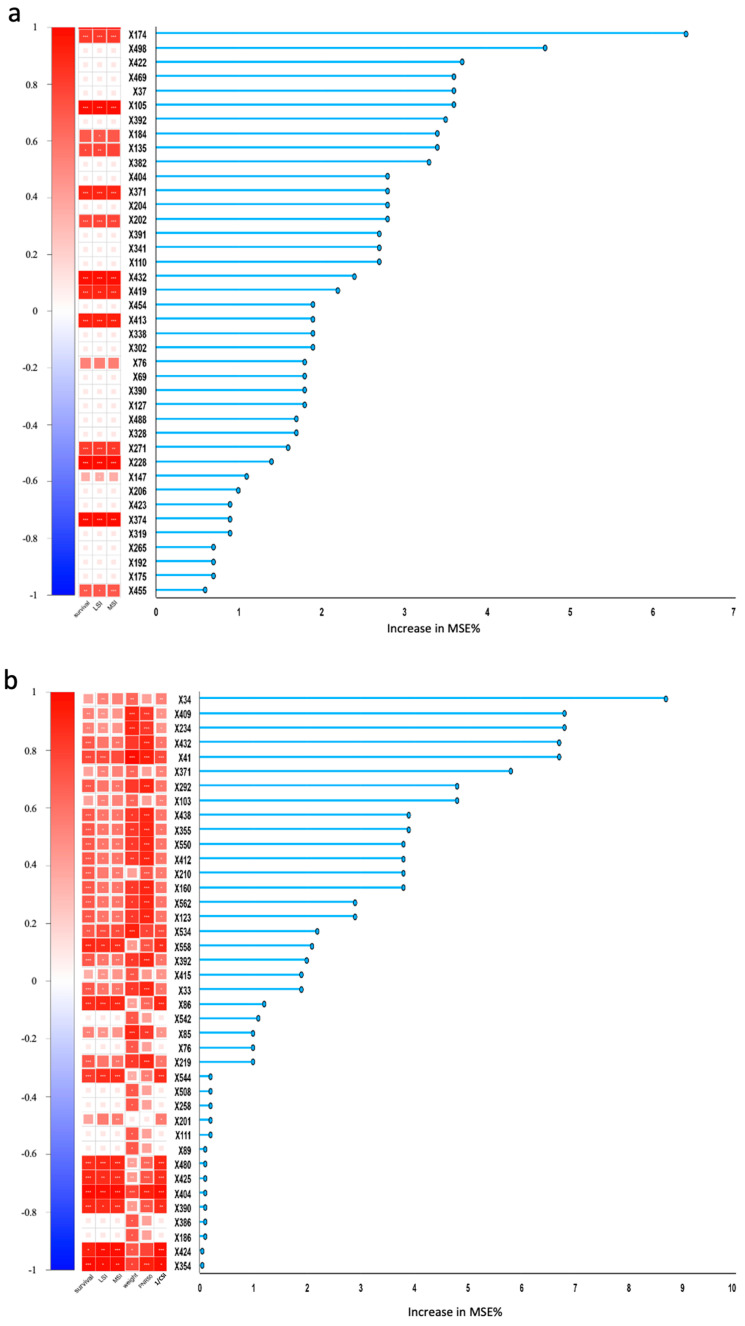
Top 40 predicted survival rate-associated species in *Scylla paramamosain* (**a**) Z3 and (**b**) Z5. Regression-based random forest algorithm in R was used. Microbial correlations with larval growth performance indices are presented as survival rate, molting synchronicity indices (MSIs), larval stage indices (LSIs), weight, point of no return when 50% mortality (PNR50), and the reciprocal of the cumulative stress index (1/CSI) indicative of sudden salinity drop. X plus strain number represents different species. The squares in the heatmap represent correlations, with positive correlations in red and negative correlations in blue, and the depth of color indicates the strength of the correlation. In each box, a different number of “*” symbols is used to indicate the significance level: * *p* < 0.05; ** *p* < 0.01; *** *p* < 0.001.

To identify the growth performance-associated probiotics within key developmental stages of *Scylla paramamosain* zoea, a random forest analysis was performed using the survival rate (the priority goal of larviculture) as the primary outcome (Figure 8). The random forest model was highly effective, with R^2^ values of 0.981 for the training set and 0.933 for the test set, indicating good fit. The explained variance scores (EVSs) were 0.985 and 0.978, respectively, indicating a strong explanation for the fluctuation in the data. Among the predicted strains in Z3, the abundances of X174 (*Halobacteriovorax* unclassified), X105 (*Confluentimicrobium* unclassified), X371 (*Rhodospirillales* unclassified), X202 (*Lactobacillus* unclassified), X432 (*Sulfitobacter* unclassified), X413 (SM1A02 unclassified), X228 (*Maritalea* unclassified), and X374 (*Rickettsiales* unclassified) were significantly and positively correlated with survival, LSI and MSI (*p* < 0.001). X105 (*Confluentimicrobium* unclassified) was the core strain in all larval stages with high abundance (Z > 0), whereas X202 (*Lactobacillus* unclassified) was a hub node connecting different larval stages. In Z5, the abundance of X404 (*Salinovum* sp.) was significantly positively correlated with the survival rate, LSI, MSI, final body weight, PNR50, and 1/CSI (*p* < 0.001). X480 (*Sneathiella* unclassified), X425 (*Rhodobacterales* unclassified), X544 (uncultured *Phaeobacter* sp.), X86 (*Clostridia* UCG-014 unclassified), X41 (*Arenibacter* unclassified), and X210 (*Lacticaseibacillus* unclassified) were also significantly correlated with several growth performance indices. Among these genera, X425 (*Rhodobacterales* unclassified) was the dominant genus in Z1 (Figure 4c). Functionally, X210 (*Lacticaseibacillus* unclassified) can also be identified as *Lactobacillus*, whereas X86 (*Clostridia* UCG-014 unclassified) might be a butyric acid producer.

A deeper analysis of the individual bacteria via LEfSe analysis (Figure S6) revealed that X105 (*Confluentimicrobium* unclassified), X432 (*Sulfitobacter* unclassified), and X202 (*Lactobacillus* unclassified) in Z3, as well as X105 (*Confluentimicrobium* unclassified), X432 (*Sulfitobacter* unclassified), and X425 (*Rhodobacterales* unclassified) in Z5, were enriched by PM to achieve the best overall growth performance, which were also listed among the top features predicted by the random forest.

**Figure 9:**
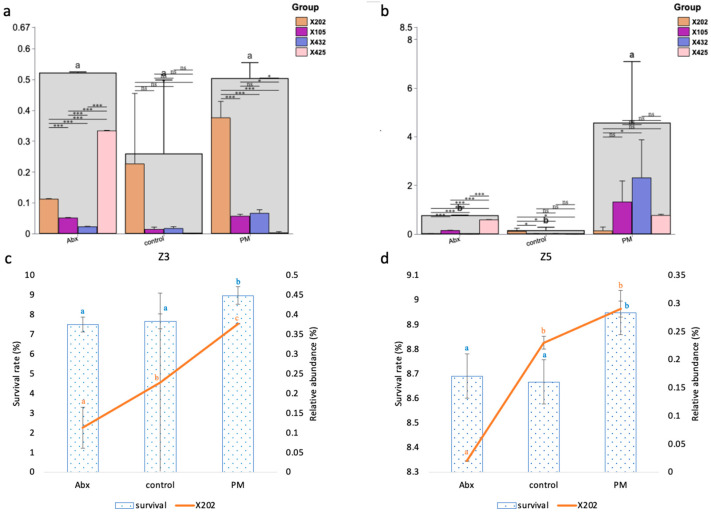
Relative abundance of candidate growth-promoting prebiotics. In *Scylla paramamosain* (**a**) Z3 and (**b**) Z5, four candidates were determined. X202 (second Y axis) and the survival rates (first Y axis) of *Scylla paramamosain* (**c**) Z3 and (**d**) Z5 were compared in the prebiotic mixture (PM), antibiotic (Abx), and control groups. X plus strain number represents different species. Mother bars represent the total relative abundance of sub-bars (candidate probiotics) in that category. Different letters above a bar/line of the same color represent *p* < 0.05, denoting a statistically significant difference. * *p* < 0.05; *** *p* < 0.001.

Therefore, random forest, Pearson correlation, and LEfSe analyses simultaneously identified four growth-promoting bacteria for use as potential probiotics in *Scylla paramamosain* zoea. They were X105 (*Confluentimicrobium* unclassified), X432 (*Sulfitobacter* unclassified), X202 (*Lactobacillus* unclassified), and X425 (*Rhodobacterales* unclassified), whose relative abundances are shown in Figure 9. In Z3, the four candidate probiotics together accounted for approximately 0.5% of the total relative abundance, of which the abundance of X425 in the Abx group was the highest (*p* < 0.001), and the abundance of X202 in the PM group was the highest (*p* < 0.001). In Z5, the proportions of the four candidates in the PM treatment (>4%) were significantly greater than those in the control and Abx treatments (*p* < 0.05). The abundance of X425 in the Abx group was the highest (*p* < 0.001), and the abundance of X202 in the control group was the highest (*p* < 0.05). Among the four candidates, X202 was the strain with the highest degree of original colonization detected in the control group. The original colonization percentage of X202 in Z3 and Z5 was approximately 0.25%, indicating that it might be an endogenous strain. In both Z3 and Z5, the abundance of X202 gradually increased from the Abx group to the control group to the PM group (*p* < 0.05), which was in concert with the increasing trend of the survival rates.

## 4. Discussion

For mariculture, the production of high-quality larvae is a bottleneck. For many species, larval survival remains as low as 5–15%, suggesting that larviculture techniques are not yet ideal. Abundant evidence indicates that larval viability is largely determined by larvae–microbiota interactions [43]. Microbial colonization is an important process in larvae and a critical stage determining host health later in life [4]. Here, we tracked the establishment of *Scylla paramamosain* zoea microbiota and identified potential growth-promoting probiotics, as discussed below.

### 4.1. Dynamics of Microbiome Diversity in Scylla paramamosain Larviculture

As shown in Figure 1, the number of OTUs in *Scylla paramamosain* zoea ranged from approximately 300–350, and the number of OTUs in water ranged from approximately 300–700. The median number of OTUs in rotifers was approximately 400, similar to the 338 ± 87 OTUs reported in another study [44], whereas the median number of OTUs in Artemia was approximately 300, similar to the number of OTUs in Artemia for *Macrobrachium rosenbergii* consumption [45]. We found that the microbial community richness in water was greater than that in zoea and live feed, and the microbial community diversity in water and live feed was greater than that in zoea. Similarly, the community richness and diversity were greater in *Macrobrachium rosenbergii* culture water than in larvae and feed [45]. Other studies have also shown that the bacterial composition of culture water was more complex than that of larvae and feed [5,46,47].

The species richness and diversity of the early-stage zoea (Z1–Z2) were relatively greater than those of the late-stage zoea (Z3–Z5). A similar study revealed that the microbial diversity of *Macrobrachium rosenbergii* decreased significantly with developmental stage [2]. The alpha diversity of rainbow trout (*Oncorhynchus mykiss*) zoea decreased significantly with age [48]. The alpha diversity of *Penaeus indicus* increased significantly from egg to zoea and stabilized gradually in the later stage [3]. The Chinese mitten crab (*Eriocheir sinensis*), zebrafish (*Danio rerio*) [46], and green guppy (*Nothobranchius furzeri*) [49] all presented the highest diversity of gut microbiota in larvae, which decreased with age. The onset of feeding might be responsible for the colonization of multiple groups of microorganisms [4]. Therefore, this could explain the greater alpha diversity of the early-stage larvae (Z1–Z2) as soon as the mouth opened, whereas the diversity of the late-stage larvae (Z3–Z5) gradually stabilized. This was also observed in fish larvae, where the species richness increased by 1.7-fold upon the first ingestion of food but did not change significantly at subsequent time points [5]. In terms of beta diversity, as shown in Figure 2, Z1 diverged from the other four periods (Z2–Z5) in our study, which was similar to the pattern in *Macrobrachium rosenbergii,* where the microbial structure of Z1 differed significantly from that of Z2–Z5, and there were similarities between Z2–Z4 [2].

Additionally, we observed similar patterns of alpha diversity in the zoea and water samples that fluctuated throughout the developmental stages, both of which first decreased but then increased. The richness and diversity of water samples also tended to decrease, followed by an increasing trend in *Macrobrachium rosenbergii* larviculture [45]. A simultaneous change in the alpha diversity of larvae and water has also been reported for *Macrobrachium rosenbergii* [45] and seabream [5]. This trend was also supported by the beta diversity results, which revealed that the microbial structure of the stage III water (W3) was the most similar to that of the zoea, followed by W4 and the rotifer.

### 4.2. Origins and Factors Shaping the Host Microbiome

To evaluate the priority effect, i.e., the order and timing of species arrival, we analyzed the relative contribution of early-stage microbiome to the later-stage microbiome. The microorganisms in Z2–Z5 were mainly retained by the larvae from the previous period, with a sudden decrease in the proportion of Z2 microbiota in Z3, which might be due to a change in feed from rotifers (Z2) to Artemia (Z3). In other studies, the transformation of feed also led to changes in the microbiome [3,5,50]. The succession of the microbiota of Z3–Z5 in our study gradually increased with stage, with up to 88% of the Z5 microbiome originating from Z4, which was almost different from water and feed, indicating the stabilization of the microbial structure. Research has also revealed that by the 21st day of development, the microbial community of *Macrobrachium rosenbergii* gradually stabilized and was no longer contributed by water or feed [45].

The contribution of water and feed to the microbiome of the zoea varies depending on the developmental stage [51]. Figure 3 showed that the contribution of rotifers to the microbial community of Z1–Z2 was greater than that of water. Research has also reported that, compared with water, fish larvae have more OTUs that are shared with feed [5]. However, one study reported that 35% of the mud crab Z1 microbiome was sourced from water, suggesting that the majority of the communities were from water rather than live feed [32]. However, this study generated a proportion of unknown sources as high as 58.8%, whereas in our study, the percentage of unknown sources of Z1 was only 13%. This may be explained by discrepancies in detection accuracy or different DNA extraction abilities of the reagent kits, which led to some bacteria from live feed not being properly identified. Certainly, these unknown proportions might also represent vertical transmission from the parent microbial community [45] or from the water in the spawning pool, since female *Scylla paramamosain* completes the entire embryonic development process and directly hatches Z1. Figure 3 also shows that the contribution of water to the microbial community in Z3–Z4 was greater than that on the microbial community in Artemia. Many studies have also reported the contribution of water to the microbial community in larvae [12,32,45,47]. These results can be explained by the formation of the larval oral cavity as the larvae grow, where water can be directly absorbed by the larvae, influencing the formation of microorganisms in late-stage larvae in particular [12].

Therefore, we suggest that the microbial communities in *Scylla paramamosain* Z1 and Z3 are mostly sourced from the environment and that the hygiene of rotifers and the disinfection of water should be key concerns, whereas the optimal timing for microbial regulation might be missed when larvae develop to Z5.

### 4.3. Core and Stage-Associated Taxa

Figure 4 showed that *Proteobacteria* was the most important phylum in *Scylla paramamosain* zoea, possibly because of its carnivorous nature. Z1 contained the most *Proteobacteria*, whereas Z2 to Z5 gradually contained more *Firmicutes*, *Bacteroides,* and *Verrucomicrobia*. *Firmicutes*, *Proteobacteria*, and *Bacteroides* were also the largest phyla in *Macrobrachium rosenbergii* larvae [2]. *Proteobacteria* and *Bacteroides* are characteristic phyla in marine organisms [5,34], whereas the abundance of *Firmicutes* in the fish gut microbiota increases with age [5]. In the intestine of adult *Scylla paramamosain*, previous studies revealed that *Firmicutes*, *Proteobacteria*, and *Bacteroides* were also core phyla [52,53,54]. The *Bacteroides*/*Firmicutes* ratio increased with larval development and was significantly greater at Z4 and Z5 than at Z1–Z3 (*p* < 0.05). The *Bacteroides*/*Firmicutes* ratio in Chinese mitten crabs fed only animal meat was reportedly lower than that in omnivores and vegetarians [17]. Therefore, the role of *Firmicutes* and *Bacteroides* in the growth and development of *Scylla paramamosain* zoea could be related to dietary preference and the inclusion of diverse food types in older stages.

In *Scylla paramamosain* zoea, *Moraxellaceae* was the most dominant family, whereas *Psychobacter* was the predominant and core genus (Figure S2). Previous studies have shown that the core genus in male adult *Scylla paramamosain* was also *Psychobacter* [52]. *Psychobacter* (family *Moraxellaceae*) isolated from the intestines of *Seriola Lalandi* [55] and Atlantic cod [56] have shown antibacterial effects, and *Psychobacter* (family *Moraxellaceae*) isolated from the digestive tract of flounder can produce essential fatty acids for growth [57], indicating a possible function in immunity and lipid metabolism for larval development for all growth stages. The topological properties shown in Figure 5 suggested that the *Lactobacillus* genus was the most connected node throughout the developmental stages, which might be the main driver of the stage-associated changes in the microbiota. Nevertheless, the contribution of other co-occurring genera revealed in the network could also be of fundamental importance.

### 4.4. Effects of Microbial Intervention on Larval Growth Performance

The use of probiotics and antibiotics has broad applications in the study of the effects of gut microbes [58]. For example, changes in the gastric microbiome were induced with antibiotic and probiotic mixtures to examine the effects of gut bacteria on the fecal fatty acid profile [10]. An antibiotic-treated zebrafish model was used to determine the role of the gut microbiota in lipid metabolism [59]. To investigate the effects of microbes on the growth and development of zebrafish larvae, mixed probiotics and antibiotics have been used to alter environmental microbial populations [31]. Here, we applied three groups of *Scylla paramamosain* zoea with enhanced (PM), normal (control), and reduced (Abx) microbial communities, which was the first study to compare the effects of PM and Abx on *Scylla paramamosain* zoea. As we hypothesized, Abx decreased the microbial richness and diversity in the zoea (*p* < 0.05), whereas PM increased the microbial richness and diversity in Z5 (*p* < 0.05) despite the first decrease in Z3 (Figure S4). The PCoA map in Figure S5 showed that PM Z5, Abx Z3, and Abx Z5 were far from the control group. This finding was consistent with the study of *Litopenaeus vannamei*, in which PCA plots revealed a significant shift in the microbial structure of the probiotic group [60]. The growth and development of *Scylla paramamosain* zoea were significantly affected by different microbiomes. Figure 6 showed that the survival rates of Z3, Z4, and Z5 in the PM treatment were significantly greater than those in the Abx treatment and the control (*p* < 0.05). However, the survival rates of Z2 did not improve in the PM group, which might be caused by the delayed colonization of probiotics at the early stage of treatment, thus making it less effective than antibiotics. We observed that the survival rate increased with molting synchronicity to some degree, which might be due to the reduction in competition and cannibalism between larvae [36]. However, the final weight of Z5 in the PM and Abx groups was not greater than that in the control group (Figure 7). Other studies have shown that *Firmicutes*, although potential probiotics, were inversely associated with body length, width, and weight in Chinese mitten crabs [26]. The stress tolerance of the PM and Abx groups was greater than that of the control in the face of a sudden decrease in salinity (Figure 7). Other studies reported that the growth of *Litopenaeus vannamei* larvae was accelerated by the addition of probiotics to water, and when the larvae were suddenly exposed to freshwater or 60‰ sea water, the cumulative mortality of probiotics was significantly lower than that of the control [61]. However, in terms of hunger tolerance, Abx was superior but PM was weaker than the control (Figure 7).

### 4.5. Growth-Promoting Probiotics

Our intervention revealed that the growth traits of the larval microbiome could be affected. Therefore, we further analyzed the associations between the dynamics of the microbiome and larval growth performance via random forest (Figure 8), Pearson correlation (Figure 8), and LEfSe analyses (Figure S6) to identify candidate probiotics that promote the growth and development of *Scylla paramamosain* zoea. The random forest model is a new and highly flexible method that uses multiple trees for training and prediction via a machine learning algorithm [62]. The latest study used a random forest model to predict the gut microbiota associated with human constipation [63]. A random forest was also used to identify intestinal pathogenic microorganisms associated with enteritis in premature infants [64]. Using random forest and Spearman correlations, another study predicted that probiotics were positively correlated with bile acids and short-chain fatty acids in the gut of ruminants [65].

Our study identified four candidate probiotics, as shown in Figure 9. Among them, three candidate probiotics, X425 (*Rhodobacterales* unclassified), X432 (*Sulfitobacter* unclassified), and X105 (*Confluentimicrobium* unclassified), belong to the order *Rhodobacterales* [66]. Research has shown that the order *Rhodobacterales* is associated with lipid metabolism in larvae [67]. *Rhodobacterales* can also synthesize antibiotics, auxin, or vitamins to increase growth performance [68]. The potential probiotics reported under the order of *Rhodobacterales* [69] to promote growth already include the genera *Roseobacter* [70] and *Rhodobacter* [71]. X425 was an unclassified *Rhodobacterales* strain that was more dominant in the Abx group than in the other candidates in both Z3 and Z5 (*p* < 0.001). In Z5, the abundance of X425 was significantly positively correlated with survival rate, LSI, MSI, final body weight, PNR50, and CSI. The second candidate, X432, was an unclassified *Sulfitobacter* with a greater abundance in the PM group of Z5 than the other candidates did. The abundance of X432 in Z3 was significantly positively correlated with survival rate, LSI, and MSI, whereas its abundance in Z5 was significantly positively correlated with survival rate, MSI, PNR50, and CSI. The genus *Sulfitobacter* is the core genus in sea cucumber that promotes growth performance [72] and enhances stress resistance [73]. *Sulfitobacter* also exhibited strong antibacterial effects against *Vibrio* to improve survival [74]. Third, X105 was an unclassified *Confluentimicrobium*, a core strain present in all the zoea stages, whose abundance in Z3 was significantly positively correlated with survival rate, LSI, and MSI. The *Confluentimicrobium* genus has been reported to synthesize antibiotics [75] to improve growth and development.

On the other hand, candidate X202 (*Lactobacillus* unclassified) was a hub node connecting Z1 and Z3, and Z3 and Z5. It was significantly positively correlated with survival rate, LSI, and MSI in Z3 (*p* < 0.001), whose abundance was consistent with the change in survival rate in both stages (Figure 9). Lactic acid bacteria are commonly used as probiotics in aquatic larviculture [69] to improve the feed conversion ratio and nutrient utilization efficiency [31]. *Lactobacillus* spp., *Leuconostoc mesenteroides*, *Lactococcus lactis*, *Enterococcus* spp., *Pediococcus acidilactici*, and *Pediococcus pentosaceus* have been used as probiotics to increase immunity, increase disease resistance, regulate the gut microbiota, and compete with pathogens [76]. In a study of adult mud crabs, two strains of lactic acid bacteria improved weight, specific growth rate, and immunity [76]. The dominance of *Lactobacillus* in the gut of mud crabs significantly increased the functional pathways of immunity and lipid metabolism, reduced the pathways involved in infectious diseases, and promoted the growth of crabs [27]. Studies in other crabs have shown that the addition of *Lactobacillus* significantly increased the survival of blue swimming crab larvae and enhanced protease and amylase activity to promote growth [77,78]. In shrimp, three candidate strains of *Firmicutes* might be associated with the body weight of *Litopenaeus vannamei* [22]. In fish, lactic acid bacteria can continuously improve the survival of zebrafish larvae, promoting early differentiation and improving intestinal function [31].

Intriguingly, there was a discrepancy between the predictions of Z3 and Z5. X202 and X105 were candidate probiotics in Z3, whereas X425 was a candidate probiotic in Z5. Only X432 was the candidate shared in both stages. Nevertheless, the various roles of these candidates make them valuable for further research.

### 4.6. Strengths and Limitations

In this study, we did not dissect the intestinal tract of the zoea but rather extracted DNA from the intact zoea. Although the zoea were washed with sterile water, microbial analysis could also reveal bacteria on the skin mucus. However, with the growth of the zoea and the increase in exogenous feeding, gut bacteria constitute a major part of the total microbiota. In addition, the sequencing results revealed unclassified bacteria, some of which were key OTUs associated with zoea, indicating the novelty of the *Scylla paramamosain* zoea microbiota. Yet, it is worth considering that 16S rRNA gene amplicon sequencing is a highly sensitive bacterial community analysis method; even dead bacteria can be detected with sufficient purity. Although this technique cannot exclude bacteria that have died before sampling, their existence at specific time points has been indicated. Of course, further research with larger sample sizes can improve the reliability of the results. Second, this study used SourceTracker analysis to estimate the source of the microbial community on the basis of correlation, which did not directly prove the transfer of microorganisms from the external environment to larvae. Third, the composition of the microbiome may be influenced by either environmental factors or individual variation. We collected the same mother crab from the same batch of zoea with the same genetic background and breeding conditions before microbial intervention. Therefore, we hypothesized that there was no difference in the microbial composition at the start of the experiment. When PM was used, we did not consider the synergy of bacterial combinations. When antibiotics are used, the analysis does not consider the direct effects of antibiotics on the host or the effects on antibiotic-resistant bacteria [79]. Moreover, our study did not consider diverse dosages, ignoring probiotics that only work at specific levels. In addition, it is necessary to isolate candidate probiotics and feed them to larvae to prove their function in growth and development. Based on the link between specific microbial taxa and growth performance, experimental validation of causal evidence was performed. The validation results will be in our next article about a novel strain X202 (*Lactobacillus* unclassified) of the genus *Lactobacillus* which was selected as the screening target. Further in vitro measurements were conducted on the expression of extracellular polysaccharides, pH tolerance, high salt tolerance, bile salt tolerance, hydrophobic properties, and the enrichment efficiency, while its inhibitory effect on the pathogen *Vibrio harveyi* were evaluated in vivo. Finally, *Lactobacillus paracasei* LBS1 (GenBank access number PQ122029) was screened from *Scylla paramamosain* zoea. Growth and development at 6 h and 24 h after feeding LBS1-enriched biological feed was observed, and comparative transcriptome analysis was also performed. The results showed that continuous application of LBS1-enriched feed during the development of *Scylla paramamosain* zoea can promote their growth performance, significantly improve their survival rate, development speed, and tolerance to osmotic stress. However, LBS1 showed no significant advantage in molting synchrony, final weight, and hunger tolerance compared to the control group. Further studies should gain greater insight into the mechanisms of how gut microbes and probiotics affect larval growth and development.

## 5. Conclusions

In summary, the present study profiled the microbiomes of *Scylla paramamosain* and the shifted contributions of live feed and water to bacterial colonization across all zoea stages. We observed decreased microbial diversity and increased priority effects with developmental stage. *Psychobacter* was the predominant and core genus, whereas *Lactobacillus* was the hub genus connecting developmental stages. Enhanced (PM), normal (control), and reduced (Abx) microbiomes revealed associations between different microbiomes and stage-dependent growth traits. By combining machine learning and traditional bioinformatic analysis, we identified four candidate probiotics that promote growth performance, belonging to *Rhodobacterales*, *Sulfitobacter*, *Confluentimicrobium*, and *Lactobacillus*, respectively. Our study answered several key ecological questions related to the early life stages of *Scylla paramamosain* zoea microbiome, and provided a theoretical basis for further exploration of the role of probiotics aimed at improving larviculture techniques to obtain high yields in practice.

## Data Availability

Data are contained within the article or Supplementary Material. The dataset of original taxonomic classifications is available on request from the authors.

## Supplementary Materials

The following supporting information can be downloaded at: https://www.mdpi.com/article/10.3390/microorganisms12122457/s1, Figure S1: Rarefaction curve analysis of (**a**) trial 1 and (**b**) trail 2; Figure S2: Heatmap of microbial composition of *Scylla paramamosain* zoea stages I (Z1), II (Z2), III (Z3), IV (Z4), and V (Z5) at the species level; Figure S3: Microbiome compositions of probiotics mixture-enriched live feeds; Figure S4: Alpha diversity represented by observed OTUs of *Scylla paramamosain* (**a**) zoea stage III (Z3) and (**b**) stage V (Z5) and Simpson index of (**c**) zoea stage III (Z3) and (**d**) stage V (Z5) in the control (con), probiotic mixture (PM), and antibiotics (Abx) groups; Figure S5: Principal coordinates analysis based on Bray–Curtis distance of *Scylla paramamosain* zoea stage III (Z3) and V (Z5) in the control (con), probiotic mixture (PM), and antibiotics (Abx) groups; Figure S6: LEfSe analysis between prebiotics mixture (PM), antibiotics (Abx), and control groups at the species level in *Scylla paramamosain* zoea (**a**) Z3 and (**b**) Z5.
