# Supplementary material for: Longitudinal Microbiome Investigations Reveal Core and Growth-Associated Bacteria During Early Life Stages of Scylla paramamosain
Source: Microorganisms. 2024 Nov 29;12(12):2457. doi: 10.3390/microorganisms12122457 (PMC11678816; doi:10.3390/microorganisms12122457)
Supplement: Supplementary file 1 [file microorganisms-12-02457-s001.zip › microorganisms-3322975-supplementary.pdf]

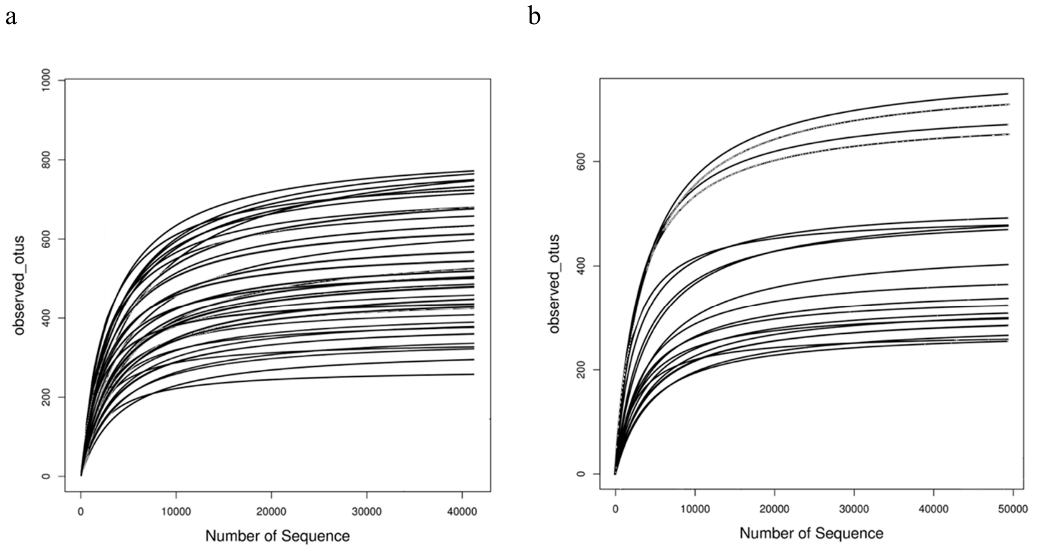

Figure S1 Rarefaction curve analysis of (**a**) trial 1 and (**b**) trail 2

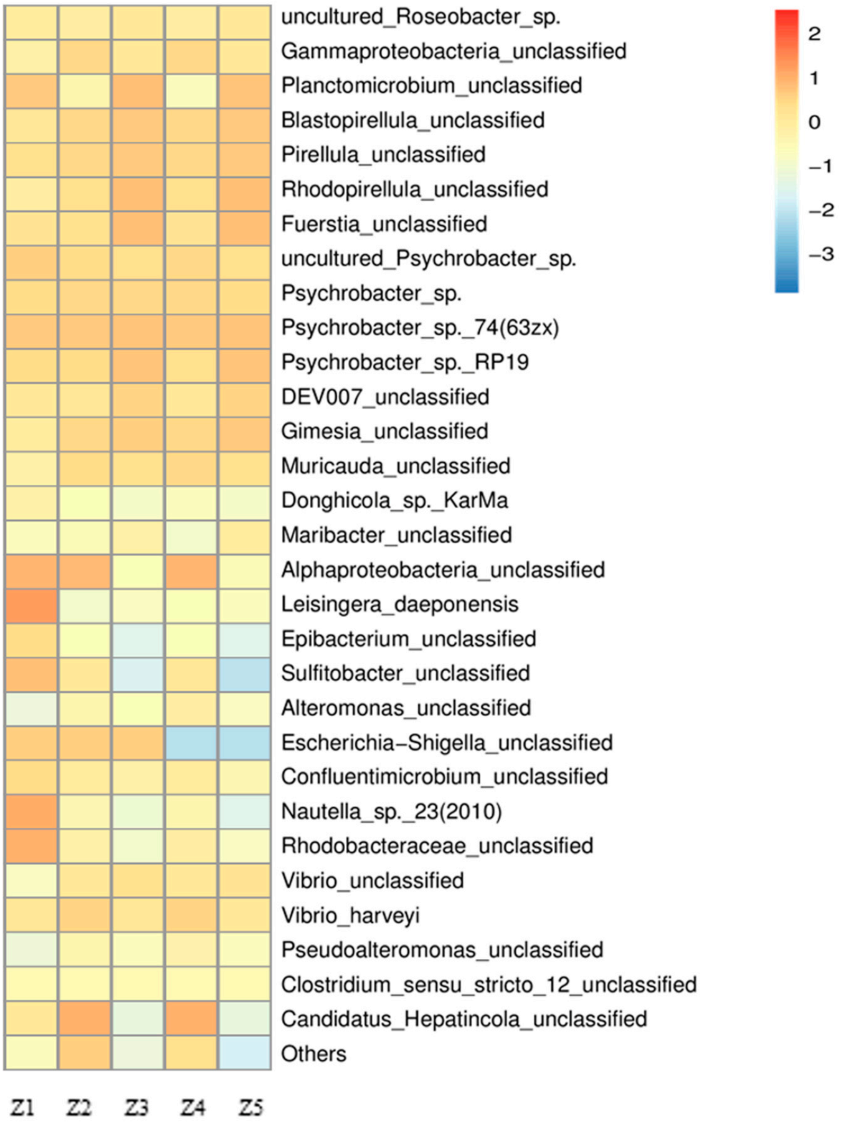

Figure S2 Heatmap of microbial composition of *Scylla paramamosain* zoea stages I (Z1), II (Z2), III (Z3), IV (Z4), and V (Z5) at the species level. The community composition data of the top 30 relative abundances at the species level based on the abundance distribution of taxonomic units or the similarity between samples were presented. This heatmap underwent Z-value conversion, and the gradient color from blue to red reflected the change in abundance from low to high.

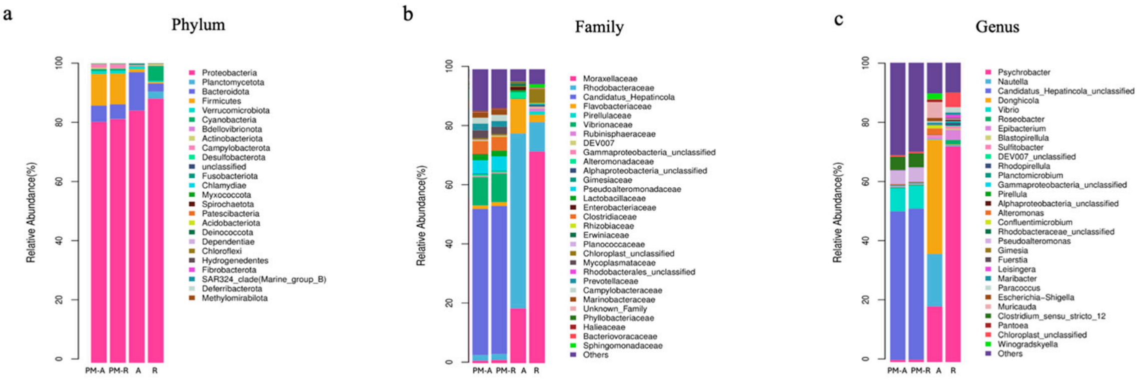

Figure S3 Microbiome compositions of probiotics mixture enriched live feeds. Stacked bar charts showed top 30 of probiotics mixture (PM) enriched rotifers (R) and artaemia (A) compared with untreated rotifers (R) and artaemia (A) at the (**a**) phylum, (**b**) family and (**c**) genus levels.

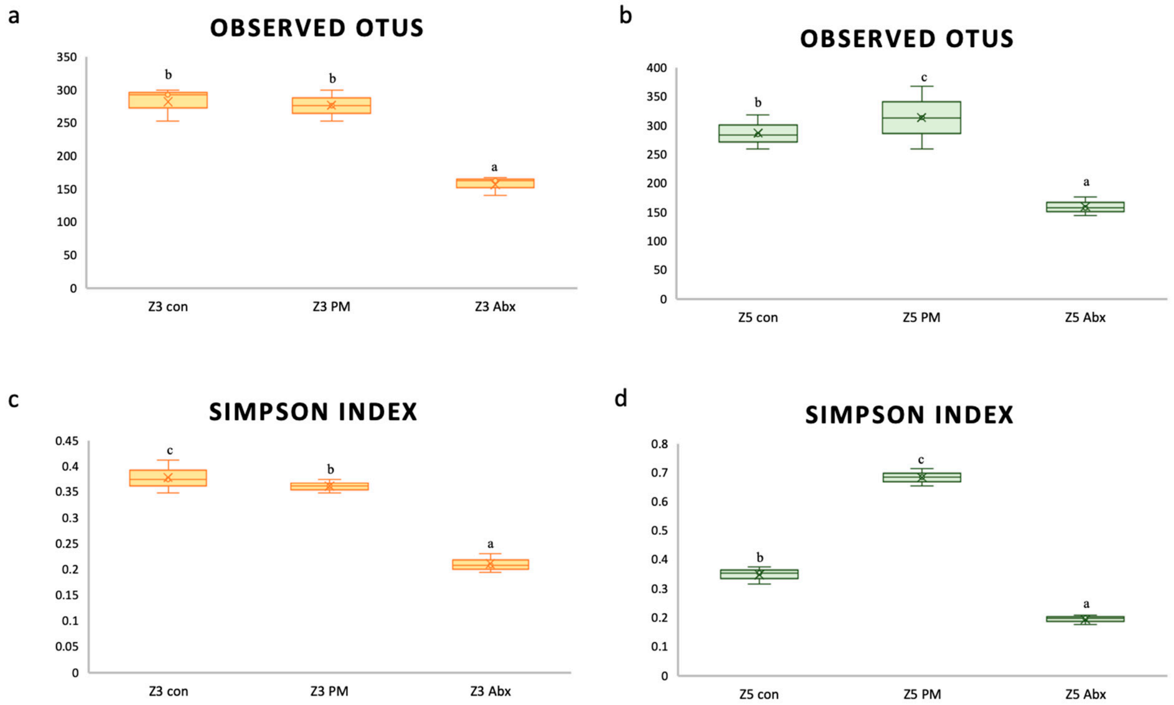

Figure S4 Alpha diversity represented by observed OTUs of *Scylla paramamosain* (**a**) zoea stage III (Z3) and (**b**) stage V (Z5) and simpson index of (**c**) zoea stage III (Z3) and (**d**) stage V (Z5) in the control (con), probiotic mixture (PM) and antibiotics (Abx) groups. The number of OTUs determines species richness, while the Simpson index determines species diversity. Different letters above box represent $P<0.05$ denoting statistically significant difference. $n = 600$.

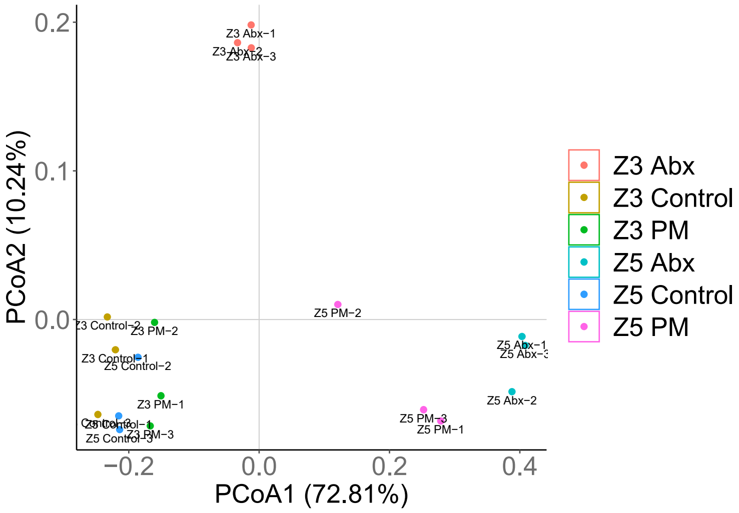

Figure S5 Principal Coordinates Analysis based on Bray_Curtis distance of *Scylla paramamosain* zoea stage III (Z3) and V (Z5) in the control (con), probiotic mixture (PM) and antibiotics (Abx) groups.

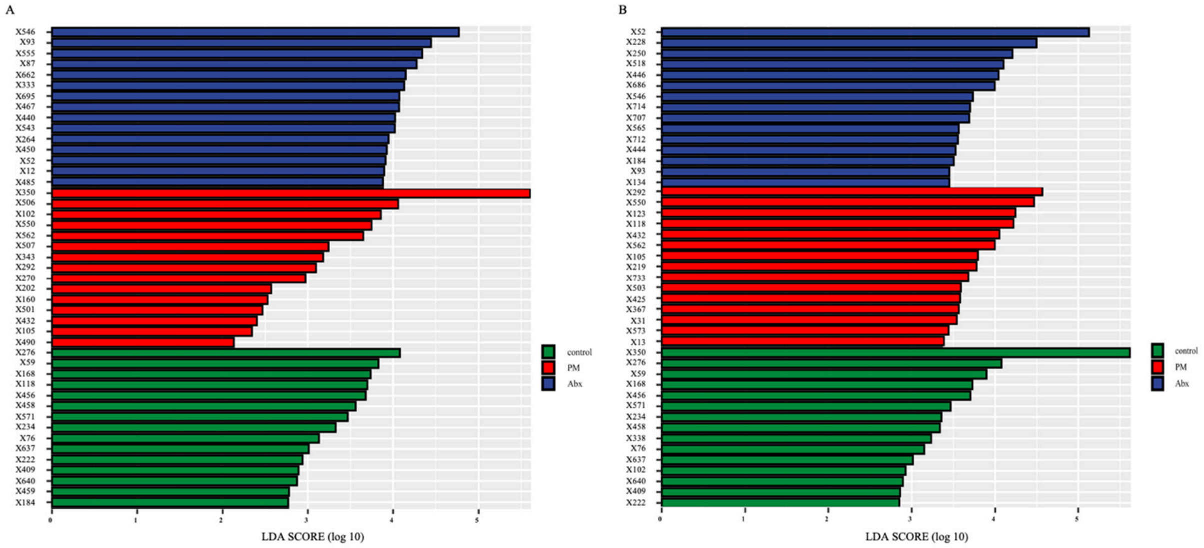

Figure S6 LEfSe analysis between prebiotics mixture (PM), antibiotics (Abx) and control groups at the species level in *Scylla paramamosain* zoea (**a**) Z3 and (**b**) Z5. X plus strain number represents different species. Linear discriminant analysis (LDA) was used to obtain the top 15 bacterial strains with the highest LDA scores in each treatment group as the differential bacterial strains.
